# Development and application of a quality evaluation scale for ERAS health education in gynecologic malignancies

**DOI:** 10.3389/fonc.2026.1858321

**Published:** 2026-06-30

**Authors:** Jianmin Feng, Lu Wang, Hanlin Yang, Weijie Tian, Yuan Gong, Yan Zhu, Dan Zi

**Affiliations:** 1 The Key Laboratory of Environmental Pollution Monitoring and Disease Control, Ministry of Education, School of Public Health, Guizhou Medical University, Guiyang, Guizhou, China; 2 Department of Gynecology, Guizhou Provincial People’s Hospital, Guiyang, Guizhou, China

**Keywords:** Delphi method, enhanced recovery after surgery (ERAS), gynecologic malignancies, health education, quality evaluation

## Abstract

**Aims:**

To develop a quality evaluation scale for ERAS health education in gynecologic malignancies and examine its reliability and validity, providing a dedicated assessment tool for clinical health education quality management that complements—rather than replaces—ERAS clinical compliance audits.

**Methods:**

Guided by the Donabedian structure-process-outcome model, an initial item pool was established through literature review and semi-structured interviews. Two rounds of Delphi consultation with 18 experts were conducted to screen items. A supporting implementation and evaluation protocol was developed simultaneously, specifying delivery agents, timing, formats, evaluation methods, and feedback-improvement mechanisms. A total of 116 perioperative patients with gynecologic malignancies were enrolled for reliability/validity testing and clinical application.

**Results:**

The final scale comprises 4 primary dimensions, 22 secondary indicators, and 36 tertiary items, corresponding to admission, preoperative, postoperative, and discharge education stages, with a total score of 100. Response rates for both Delphi rounds were 100%, with authority coefficients (Cr) of 0.897–0.914. I-CVI ranged from 0.833 to 1.000 and S-CVI/Ave was 0.946. KMO was 0.821; four factors were extracted with a cumulative variance of 51.60%. Cronbach’s α for the total scale was 0.890, and test-retest ICC was 0.925. Clinical application showed a total score of 86.7 ± 8.4. Discharge education emerged as the weakest domain (scoring rate 84.3%), with all four items scoring <70% concentrated in this dimension: psychological counseling and peer support (60.0%), sexual life resumption guidance (63.0%), home medication guidance (65.9%), and home activity/nutrition/wound care (68.1%).

**Conclusion:**

The scale demonstrates good reliability and validity. As a dedicated tool for evaluating ERAS health education quality, it complements rather than replaces comprehensive ERAS compliance audits, enabling identification of weak links and continuous quality improvement. Its association with core ERAS outcomes warrants further validation through multicenter prospective studies.

## 1 Introduction

Enhanced Recovery After Surgery (ERAS), first proposed by Danish surgeon Kehlet in 1997, refers to perioperative optimization measures based on multidisciplinary collaboration aimed at reducing surgical stress responses, lowering complication rates, and shortening hospital stays (1). Over more than two decades of development, the ERAS concept has achieved remarkable results in colorectal surgery, hepatobiliary surgery, orthopedics, and other fields (2). In the field of gynecologic malignancies, the International ERAS Society has published multiple perioperative management guidelines, and domestic experts have successively developed the “Chinese Expert Consensus on Enhanced Recovery After Gynecological Surgery” (3)and the “Chinese Expert Consensus on ERAS Perioperative Nursing in Gynecology” (4), providing important guidance for clinical practice. Gynecologic malignancies (such as cervical cancer, ovarian cancer, and endometrial cancer) involve complex surgical procedures with significant trauma and intense perioperative stress responses, and patients often experience negative emotions such as anxiety and fear (5–8). Although ERAS concepts have been gradually promoted in the perioperative management of gynecologic malignancies, their effectiveness largely depends on patients’ understanding of and compliance with relevant measures, and health education is a crucial means of promoting active patient participation (9). However, current clinical practice still suffers from problems such as unsystematic content, unreasonable implementation timing, single format, and lack of outcome evaluation in health education, affecting its practical effectiveness (10, 11). Currently, there is a lack of specific evaluation tools for the quality of ERAS health education for gynecologic malignancies in China, making it difficult to conduct systematic quantitative assessments. Based on this, this study employed the Delphi method to construct a quality evaluation scale for ERAS health education in gynecologic malignancies, and conducted preliminary clinical application and effect evaluation, aiming to provide a reference for clinical practice.

It should be noted that the successful implementation of ERAS depends on multi-dimensional quality assurance, including clinical compliance (e.g., goal-directed fluid therapy, early urinary catheter removal, multimodal analgesia execution), multidisciplinary team adherence, patient compliance, and health education quality. Although audit tools targeting ERAS clinical compliance—such as the ERAS Interactive Audit System (EIAS)—are already available, structured evaluation tools specifically designed for patient health education quality remain relatively limited. Patient education serves as the critical bridge between ERAS clinical measures and patient adherence behaviors: without patient understanding of measures such as early ambulation, preoperative carbohydrate loading, and multimodal analgesia, even the most refined clinical pathway can hardly translate into optimal recovery outcomes. Accordingly, the present study aims to develop a dedicated tool for evaluating ERAS health education quality that complements—rather than replaces—comprehensive ERAS compliance audits, jointly supporting the ERAS quality assurance system.

## 2 Materials and methods

### 2.1 Study setting and institutional ERAS protocol

This study was conducted in the gynecologic oncology ward of Guizhou Provincial People’s Hospital. The ward implements perioperative ERAS pathways in accordance with the Chinese Expert Consensus on Enhanced Recovery After Gynecological Surgery and the international ERAS^®^ Society guidelines. Major elements are summarized in **Table 1**.

**Table 1:** Major elements of the institutional ERAS protocol in the gynecologic oncology ward.

Phase	Main ERAS measures
Preoperative	Preoperative education; NRS2002 nutritional screening and intervention; smoking/alcohol cessation; prehabilitation training; psychological assessment and support; 6-hour fasting and oral carbohydrate loading (300 ml) 2 hours before surgery; avoidance of routine mechanical bowel preparation; VTE risk assessment and prophylaxis
Intraoperative	Short-acting anesthetics; goal-directed fluid therapy; active warming (core temperature ≥36 °C); avoidance of routine nasogastric tube; selective use of drains; prophylactic antibiotics; multimodal analgesia
Postoperative	Liquid diet within 6 hours and gradual transition; ambulation within 24 hours; urinary catheter removal within 24–48 hours; multimodal analgesia; PONV prevention; VTE prophylaxis; wound and tube management; early rehabilitation training
Post-discharge	Home rehabilitation guidance; follow-up; continued psychological and nutritional support

The scale developed in this study specifically targets those ERAS measures that require patient understanding and cooperation, aiming to evaluate whether the nursing team effectively conveys ERAS principles and behavioral requirements to patients. This study focused solely on the quality evaluation of health education and did not collect compliance data on the above clinical measures, nor did it analyze the association between education quality and core ERAS outcomes; these limitations are discussed below.

### 2.2 Scale development

#### 2.2.1 Establishment of the research team

The research team consisted of 11 members, including 3 gynecologic oncology specialist nurses, 3 gynecologic oncology physicians, 3 nursing managers, and 2 evidence-based nursing researchers, responsible for literature retrieval, interview implementation, questionnaire design, and data analysis.

#### 2.2.2 Establishment of the initial item pool

An initial item pool was established through a combination of literature research and qualitative interviews.

1. Literature research: The research team systematically searched domestic and international databases including PubMed, CINAHL, CNKI, Wanfang, and VIP from database inception to December 2024. Chinese search terms included “enhanced recovery after surgery,” “gynecologic malignancies,” “health education,” and “quality evaluation”; English search terms included “enhanced recovery after surgery,” “gynecologic oncology,” “patient education,” and “quality evaluation.” The International ERAS Society’s perioperative management guidelines for gynecologic oncology and relevant domestic expert consensus were referenced to summarize the core content and quality evaluation elements of ERAS health education for gynecologic malignancies.
2. Semi-structured interviews: Using purposive sampling, interviews were conducted with 6 gynecologic oncology specialist nurses, 6 gynecologic oncology physicians, and 20 postoperative patients to understand the current status of health education implementation and quality evaluation needs. Interview data were analyzed using thematic analysis to extract items. Based on the Donabedian model as the theoretical framework, combined with the temporal characteristics of ERAS perioperative management, items were organized into 4 dimensions: admission education, preoperative education, postoperative education, and discharge education, forming an initial item pool of 54 items after research team discussion.

#### 2.2.3 Delphi expert consultation

##### 2.2.3.1 Expert selection

Experts were selected using purposive sampling. Inclusion criteria were: ① engaged in clinical gynecologic oncology, nursing, ERAS, or nursing management; ② working experience ≥10 years; ③ bachelor’s degree or above; ④ associate senior professional title or above; ⑤ voluntary participation.

##### 2.2.3.2 Expert consultation process

Two rounds of expert consultation were conducted via email. In the first round, experts rated the importance of each item in the initial item pool using a 5-point Likert scale (1 = not important, 5 = very important), with open-ended questions for supplementary opinions. After collection, the mean importance score (mean ± SD), coefficient of variation (CV), and full-score rate were calculated. Items were retained based on criteria of mean importance ≥3.5, CV ≤0.25, and full-score rate ≥20%, and the second-round questionnaire was formed after research team discussion. In the second round, the summarized first-round results were fed back to experts for re-scoring to reach consensus.

#### 2.2.4 Scale formation and scoring

After two rounds of expert consultation, the research team systematically screened and revised the initial item pool based on the screening criteria (mean importance ≥3.5, CV ≤0.25, full-score rate ≥20%) and expert suggestions. After addition, deletion, merging, and refinement of expressions, the final scale was formed. The proportional weighting method based on expert importance ratings was used to determine the full score of each item, calculated as:

Item full score = (mean importance score of the item ÷ sum of mean importance scores of all 36 tertiary items) × 100.

The total score of the scale was set at 100 points. To facilitate clinical use, the full scores of primary dimensions were rounded (admission education 30 points, preoperative education 25 points, postoperative education 31 points, discharge education 14 points), while the full scores of secondary and tertiary items retained their original proportional values. Scoring criteria: Each tertiary item is rated on a 5-point Likert scale, corresponding to a percentage of the item’s full score: Level 1 (completely not achieved) = 20%, Level 2 (basically not achieved) = 40%, Level 3 (partially achieved) = 60%, Level 4 (basically achieved) = 80%, and Level 5 (fully achieved) = 100%. For example, for an item with a full score of 2.7 points, Level 5 = 2.7, Level 4 = 2.16, Level 3 = 1.62, Level 2 = 1.08, and Level 1 = 0.54 points. Detailed scoring criteria for all 36 tertiary items are provided in **Supplementary Material S1**.

### 2.3 Reliability and validity testing

The same 116-patient cohort was used for both reliability/validity testing and clinical application; inclusion and exclusion criteria are detailed in the Clinical Application section below.

Content validity: The 18 experts who participated in the Delphi consultation were invited to rate the content relevance of each item using a 4-point Likert scale (1 = not relevant, 2 = weakly relevant, 3 = relevant, 4 = highly relevant). I-CVI and S-CVI/Ave were calculated, with I-CVI ≥0.78 and S-CVI/Ave ≥0.90 as acceptable standards.

Construct validity: Exploratory factor analysis was used to evaluate construct validity. KMO value >0.70 and Bartlett’s test of sphericity P < 0.05 were used as criteria for suitability of factor analysis. Principal component analysis was used to extract common factors with varimax orthogonal rotation, with factor loadings >0.40 as the item retention criterion.

Reliability: Cronbach’s α coefficient was used to evaluate internal consistency reliability, with α ≥0.70 as the acceptable standard. ICC was used to evaluate test-retest reliability, with 30 randomly selected patients retested after a 2-week interval, and ICC ≥0.75 as the acceptable standard.

### 2.4 Implementation and evaluation process of health education

To ensure that scale evaluation is anchored in a clearly defined implementation pathway with observable behavioral standards, a supporting implementation and evaluation protocol was developed simultaneously.

1. Delivery agents: Primary nurses served as the core implementers; all participating nurses completed ≥8 hours of ERAS-specific training and passed standardized assessments. Attending physicians, anesthesiologists delivered specialty-specific content. Head nurses were responsible for quality supervision and continuous improvement.
2. Timing: Health education was embedded into four key time points along the ERAS pathway—admission day, 1–2 days before surgery, 24 hours postoperatively to the day before discharge, and discharge day—corresponding one-to-one with the four primary dimensions of the scale.
3. Delivery format: A multimodal approach was adopted, combining oral explanation, illustrated booklets, video demonstration, hands-on modeling, and teach-back confirmation. After each key item, patients were asked to verbally restate or physically demonstrate the content, and only after confirmed understanding did the education proceed.
4. Evaluation method: A dual-perspective design combining nurse self-evaluation and patient/family other-evaluation was used. Each tertiary item was rated on a 5-point Likert scale (1 = completely not achieved, 5 = fully achieved), corresponding to 20%–100% of the item’s full score. Quality-control nurses averaged both ratings to determine the final score.
5. Feedback and improvement mechanism: A closed-loop “education–evaluation–feedback–improvement” mechanism was established: (i) at the individual level, items not mastered by patients were supplemented by primary nurses before discharge; (ii) at the ward level, head nurses summarized weak items weekly and provided feedback during morning meetings; (iii) at the quality-control level, quarterly PDCA cycles were conducted based on cumulative scale results.

### 2.5 Clinical application

Using convenience sampling, 116 patients with gynecologic malignancies who underwent elective surgery in the gynecology department of our hospital from January to December 2025 were selected as the study subjects. Inclusion criteria: ① pathologically confirmed gynecologic malignancy; ② elective surgical treatment; ③ clear consciousness with normal communication ability; ④ voluntary participation. Exclusion criteria: ① combined with other serious diseases; ② psychiatric disorders or cognitive dysfunction; ③ withdrawal from the study. The constructed scale was used to evaluate nurses’ health education implementation (self-evaluation) and patients’ or their family members’ health education reception (other-evaluation); nurses and patients/family members independently completed their respective ratings according to the detailed scoring criteria for each item (see **Supplementary Material S1**). Scoring rates for each dimension and item were calculated to identify weak links in health education quality.

### 2.6 Statistical methods

SPSS 27.0 software was used for data analysis. Continuous data were expressed as mean ± SD, and categorical data were expressed as frequencies and percentages. P < 0.05 was considered statistically significant. Questionnaire response rate was used to reflect expert enthusiasm. The authority coefficient (Cr) was used to evaluate expert authority, calculated as Cr = (judgment basis coefficient Ca + familiarity coefficient Cs)/2, with Cr ≥0.70 as acceptable. Kendall’s W was used to evaluate the degree of consensus among experts; χ² test was applied, with P < 0.05 indicating convergence of expert opinions.

## 3 Results

### 3.1 Expert characteristics and consultation results

A total of 18 experts from 14 hospitals in 7 provinces and cities participated in both rounds of consultation. Expert characteristics are presented in **Table 2**. The response rates for both rounds were 100%. The authority coefficients (Cr) were 0.897 and 0.914 for the first and second rounds, respectively, both >0.70, indicating high expert authority. Kendall’s W for all levels of items in both rounds was statistically significant (P < 0.05), and the second-round W values were higher than those of the first round, indicating that expert opinions gradually converged with successive consultation rounds (**Table 3**).

**Table 2:** Expert characteristics (n=18).

Variable	Category	n	%
Age (years)	≤40	1	5.6
	41–49	6	33.3
	≥50	11	61.1
Gender	Male	7	38.9
	Female	11	61.1
Professional title	Associate senior	5	27.8
	Senior	13	72.2
Professional field	Nursing education	2	11.1
	Clinical nursing	2	11.1
	Nursing management	2	11.1
	Clinical medicine	12	66.7
Highest education	Bachelor's	5	27.8
	Master's	2	11.1
	Doctoral	11	61.1
Postgraduate supervisor	No	4	22.2
	Yes	14	77.8
Years of experience	11–15	1	5.6
	16–20	2	11.1
	≥20	15	83.3

**Table 3:** Analysis of expert opinion concordance.

Item level	Kendall's W	χ²	P
Primary items	0.291	15.720	< 0.001
Secondary items	0.157	59.351	< 0.001
Tertiary items	0.144	90.959	< 0.001

Kendall's W = Kendall's coefficient of concordance.

### 3.2 Scale development results

Following two rounds of expert consultation, 6 items were deleted, 8 items were added, and several items were consolidated or refined based on item selection criteria and expert suggestions. The final scale comprised 4 primary dimensions, 22 secondary indicators, and 36 tertiary items, with weighted scores assigned using proportional allocation based on expert importance ratings, totaling 100 points (admission education: 30 points; preoperative education: 25 points; postoperative education: 31points; discharge education: 14 points). Details are presented in **Table 4**.

**Table 4:** Quality Evaluation Scale for ERAS Health Education in Gynecologic Malignancies.

First-level Dimension	Second-level Indicator	Tertiary item	Importance score (±*s*)	CV	Full-score rate (%)
I-1 Admission Education (30)			5.00±0.00	0.000	100.00
	II-1 Environment, Safety and Personnel Introduction (10.7)		4.22±0.81	0.191	44.44
		III-1 Hospital environment: ward, physician office, nursing station, hot water room (2.7)	4.39±0.70	0.159	50.00
		III-2 Medical staff: attending physician, nurse, head nurse, department director (2.6)	4.33±0.69	0.159	44.44
		III-3 High-risk patient identification management (wristband, bedside identification, etc.) (2.7)	4.50±0.62	0.137	55.56
		III-4 Fall/bed fall/pressure ulcer prevention (2.7)	4.50±0.51	0.114	50.00
	II-2 ERAS Concept Overview (2.9)		4.72±0.58	0.122	77.78
		III-5 ERAS concepts and benefits (2.9)	4.78±0.43	0.122	77.78
	II-3 VTE Prevention Management (2.8)		4.78±0.55	0.115	83.33
		III-6 VTE risk assessment and prevention (2.8)	4.67±0.49	0.127	66.67
	II-4 Examination Guidance (2.7)		4.50±0.62	0.137	55.56
		III-7 Preoperative examination items and preparation (2.7)	4.44±0.78	0.155	61.11
	II-5 Preoperative Prehabilitation (8.4)		4.78±0.43	0.090	77.78
		III-8 Psychological assessment and support (2.8)	4.61±0.50	0.135	61.11
		III-9 Prehabilitation training (2.8)	4.67±0.49	0.132	66.67
		III-10 Nutritional screening and intervention (2.8)	4.61±0.50	0.135	61.11
	II-6 Disease Knowledge (2.7)		4.44±0.62	0.139	50.00
		III-11 Disease knowledge, surgical plan and fertility counseling (2.7)	4.44±0.71	0.159	55.56
I-2 Preoperative Education (25)			4.94±0.24	0.048	94.44
	II-7 Preoperative Medication and Pain Management (8.5)		4.78±0.43	0.090	77.78
		III-12 Purpose and method of preoperative medication (2.7)	4.39±0.70	0.159	50.00
		III-13 Preoperative pain assessment (2.8)	4.61±0.50	0.109	61.11
		III-14 Multimodal analgesia regimen (3.0)	4.89±0.32	0.115	88.89
	II-8 Preoperative Physical and Psychological Preparation (5.3)		4.56±0.62	0.135	61.11
		III-15 Personal hygiene and infection prevention (2.6)	4.33±0.69	0.177	44.44
		III-16 Emotional regulation and sleep management (2.7)	4.50±0.51	0.139	50.00
	II-9 Preoperative Dietary Preparation (5.7)		4.72±0.46	0.098	72.22
		III-17 Purpose of preoperative oral carbohydrates (2.9)	4.72±0.46	0.127	72.22
		III-18 Preoperative fasting, fluid restriction and bowel preparation (2.8)	4.56±0.51	0.159	55.56
	II-10 Surgical Preparation Guidance (5.7)		4.67±0.59	0.127	72.22
		III-19 Surgical and anesthesia procedures (2.8)	4.56±0.71	0.151	66.67
		III-20 Postoperative tube management and early removal (2.9)	4.83±0.38	0.079	83.33
I-3 Postoperative Education (31)			4.67±0.59	0.127	72.22
	II-11 Positioning and Activity Guidance (5.6)		4.61±0.61	0.132	66.67
		III-21 Postoperative positioning and early mobilization (2.9)	4.83±0.38	0.079	83.33
		III-22 Urination and pelvic floor function exercise (2.7)	4.50±0.71	0.155	61.11
	II-12 Postoperative Dietary Guidance (2.9)		4.72±0.46	0.098	72.22
		III-23 Early feeding and dietary transition (2.9)	4.78±0.43	0.122	77.78
	II-13 Medication and Adverse Reaction Monitoring (5.6)		4.72±0.46	0.098	72.22
		III-24 Postoperative medication guidance (2.8)	4.61±0.50	0.135	61.11
		III-25 Recognition and management of drug adverse reactions (2.8)	4.56±0.51	0.135	55.56
	II-14 Common Discomfort and Wound Management (5.5)		4.78±0.43	0.090	77.78
		III-26 Management of common postoperative discomforts (2.8)	4.61±0.50	0.135	61.11
		III-27 Wound care and infection prevention (2.7)	4.50±0.71	0.157	61.11
	II-15 Pain Management (2.9)		4.94±0.24	0.048	94.44
		III-28 Pain assessment and analgesia regimen (2.9)	4.72±0.46	0.098	72.22
	II-16 Complication Prevention (5.6)		4.83±0.38	0.079	83.33
		III-29 VTE prevention (2.9)	4.78±0.43	0.090	77.78
		III-30 Lymphedema prevention and recognition (2.7)	4.44±0.71	0.159	55.56
	II-17 Chemotherapy Guidance (if applicable) (2.7)		4.61±0.61	0.132	66.67
		III-31 Chemotherapy-related symptom monitoring (2.7)	4.39±0.78	0.177	55.56
I-4 Discharge Education (14)			4.56±0.62	0.135	61.11
	II-18 Home Life and Rehabilitation Guidance (2.7)		4.50±0.62	0.137	55.56
		III-32 Home activity, nutrition and wound care (2.7)	4.39±0.78	0.177	55.56
	II-19 Sexual Life Guidance (if applicable) (2.7)		4.50±0.71	0.157	61.11
		III-33 Sexual life resumption guidance (2.7)	4.50±0.71	0.159	61.11
	II-20 Medication Guidance (2.7)		4.67±0.59	0.127	72.22
		III-34 Home medication guidance (2.7)	4.44±0.78	0.177	61.11
	II-21 Follow-up Arrangements (2.8)		4.67±0.49	0.104	66.67
		III-35 Follow-up plan and contact information (2.8)	4.67±0.49	0.135	66.67
	II-22 Psychological Support (2.7)		4.61±0.61	0.132	66.67
		III-36 Psychological counseling and peer support (2.7)	4.39±0.70	0.209	50.00

Numbers in parentheses indicate item full scores (total = 100), derived from proportional allocation of mean importance ratings: item full score = (item mean ÷ sum of means of 36 items) × 100. Full scores for first−level items are rounded to integers for clinical use; second− and third−level items retain the original proportional values.This table presents the overall framework of the scale (first-level dimensions, second-level indicators, tertiary items, and corresponding point values). Detailed scoring criteria for each tertiary item (education standards and 5-level scoring rubrics) are provided in **Supplementary Material S1**.

### 3.3 Reliability and validity results

#### 3.3.1 Content validity

The 18 Delphi experts rated the content relevance of each scale item using a 4-point Likert scale (1 = not relevant, 2 = weakly relevant, 3 = relevant, 4 = highly relevant). Results showed that I-CVI ranged from 0.833 to 1.000 and S-CVI/Ave was 0.946, both meeting the acceptable standards (I-CVI ≥0.78, S-CVI/Ave ≥0.90), indicating good content validity.

#### 3.3.2 Construct validity

Exploratory factor analysis was conducted on the scale scores of 116 patients. The KMO value was 0.821, and Bartlett’s test of sphericity yielded χ² = 1905.75, df = 630, P < 0.001, indicating suitability for factor analysis. Using principal component analysis with eigenvalue >1 as the initial extraction criterion, 6 common factors were initially extracted with a cumulative variance contribution rate of 57.88%. Based on the scree plot and the scale’s theoretical framework, 4 common factors were retained after varimax orthogonal rotation, with a cumulative variance contribution rate of 51.60%. Factor loadings for all 36 items ranged from 0.569 to 0.796, all >0.40. The four factors corresponded to admission education (11 items), preoperative education (9 items), postoperative education (11 items), and discharge education (5 items), which was highly consistent with the scale’s four-dimensional theoretical framework (**Table 5**), indicating good construct validity.

**Table 5:** Rotated factor loading matrix of the Quality Evaluation Scale for ERAS Health Education in Gynecologic Malignancies (n=116).

Item	Factor 1 Postoperative Education	Factor 2 Admission Education	Factor 3 Preoperative Education	Factor 4 Discharge Education
Q22 (III-22)	0.763	—	—	—
Q31 (III-31)	0.749	—	—	—
Q30 (III-30)	0.745	—	—	—
Q25 (III-25)	0.741	—	—	—
Q26 (III-26)	0.705	—	—	—
Q21 (III-21)	0.689	—	—	—
Q23 (III-23)	0.687	—	—	—
Q27 (III-27)	0.684	—	—	—
Q28 (III-28)	0.662	—	—	—
Q29 (III-29)	0.657	—	—	—
Q24 (III-24)	0.608	—	—	—
Q6 (III-6)	—	0.786	—	—
Q3 (III-3)	—	0.729	—	—
Q4 (III-4)	—	0.689	—	—
Q11 (III-11)	—	0.688	—	—
Q9 (III-9)	—	0.675	—	—
Q10 (III-10)	—	0.663	—	—
Q7 (III-7)	—	0.646	—	—
Q5 (III-5)	—	0.631	—	—
Q2 (III-2)	—	0.630	—	—
Q8 (III-8)	—	0.577	—	—
Q1 (III-1)	—	0.569	—	—
Q19 (III-19)	—	—	0.757	—
Q14 (III-14)	—	—	0.756	—
Q17 (III-17)	—	—	0.753	—
Q20 (III-20)	—	—	0.697	—
Q16 (III-16)	—	—	0.683	—
Q13 (III-13)	—	—	0.682	—
Q18 (III-18)	—	—	0.674	—
Q15 (III-15)	—	—	0.654	—
Q12 (III-12)	—	—	0.647	—
Q35 (III-35)	—	—	—	0.796
Q32 (III-32)	—	—	—	0.774
Q33 (III-33)	—	—	—	0.762
Q36 (III-36)	—	—	—	0.716
Q34 (III-34)	—	—	—	0.576

#### 3.3.3 Reliability

The Cronbach’s α coefficient for the total scale was 0.890, and for each dimension ranged from 0.805 to 0.902, all >0.70. The test-retest ICC for the total scale was 0.925 (95% CI: 0.788–0.964), and for each dimension ranged from 0.883 to 0.933 (all P < 0.001), indicating good internal consistency and test-retest reliability (**Table 6**).

**Table 6:** Reliability results for each dimension of the scale.

Dimension	No. of items	Cronbach's α	Test-retest ICC (95% CI)
Admission education	11	0.879	0.883 (0.769–0.933)
Preoperative education	9	0.881	0.907 (0.827–0.945)
Postoperative education	11	0.902	0.933 (0.902–0.954)
Discharge education	5	0.805	0.907 (0.868–0.935)
Total scale	36	0.890	0.925 (0.788–0.964)

### 3.4 Clinical application results

#### 3.4.1 Participant characteristics

A total of 116 perioperative patients with gynecologic malignancies were enrolled, with ages ranging from 28 to 73 years, predominantly aged 45–59 years (54.3%). There were 75 Han Chinese patients (64.66%) and 41 ethnic minority patients (35.34%); 57 urban residents (49.14%) and 59 rural residents (50.86%). The predominant educational level was primary school or below (57.76%). Diagnoses included cervical cancer (n = 54, 46.55%), endometrial cancer (n = 56, 48.28%), and ovarian cancer (n = 6, 5.17%).

#### 3.4.2 Health education quality and content analysis of weak items

The total health education quality score for the 116 patients was 86.7 ± 8.4 (overall scoring rate 86.7%), indicating a “basically achieved” level, with approximately 13 points of room for improvement before reaching “fully achieved.” Dimension-level scores are presented in **Table 7**: admission education 26.5 ± 2.9 (88.3%), preoperative education 21.9 ± 2.5 (87.6%), postoperative education 26.5 ± 3.1 (85.5%), and discharge education 11.8 ± 2.2 (84.3%). The overall pattern indicates “relatively solid admission and preoperative education, with postoperative—and particularly discharge—education being weaker.”

**Table 7:** Health education quality scores by dimension (n=116).

Dimension	Full score (points)	Score ( $\bar{\text{x}}$±s, points)	Scoring rate (%)
Admission education	30	26.5±2.9	88.3
Preoperative education	25	21.9±2.5	87.6
Postoperative education	31	26.5±3.1	85.5
Discharge education	14	11.8±2.2	84.3
Total	100	86.7±8.4	86.7

Among the 36 tertiary items, all four items with scoring rates below 70% were concentrated in the discharge education dimension, and can be grouped into four content-level problems:

1. Widespread absence of post-discharge psychological support (psychological counseling and peer support, 60.0%): Interviews revealed that on discharge day, nurses often dismissed psychological needs with a brief “contact us if you have problems,” without proactively assessing post-discharge emotional status or providing specific referral resources such as counseling hotlines, peer support groups, or psychiatric clinics.
2. Mutual avoidance in sexual recovery counseling (63.0%): Nurses worried that “it might be inappropriate to discuss,” while patients—affected by stigma—felt uncomfortable raising the issue. Consequently, the topic was typically deferred to “whatever the doctor says,” leaving patients confused about timing of resumption and potential impact on treatment.
3. Lack of written support for home medication guidance (65.9%): Patients often left the hospital with 3–5 medications, yet instructions were largely delivered verbally without a structured written medication list, leading to missed or incorrect doses—especially among older or less educated patients—within days of discharge.
4. Home rehabilitation guidance not tailored to family settings (68.1%): Instructions remained at the level of general principles (e.g., “light diet,” “moderate activity”) without concrete, family-specific plans (e.g., daily step counts, recommended dishes, wound protection during bathing, warning signs requiring medical attention).

In contrast, items with higher scoring rates clustered around components supported by clearly defined ERAS pathways and explicit behavioral standards, such as multimodal analgesia explanation, early postoperative ambulation, preoperative carbohydrate loading, and early tube-removal education. This indicates that when educational content is embedded in clinical pathways with explicit behavioral benchmarks, nurses deliver more reliably and patients comprehend more clearly. The finding also points to a clear direction for improving discharge education—namely, redesigning it to be equally structured, checklist-based, and process-oriented.

## 4 Discussion

### 4.1 Necessity of developing the scale

The International ERAS Society has published multiple perioperative management guidelines for gynecologic oncology, with the 2023 updated version explicitly stating that the translation of guidelines into clinical practice still requires the support of systematic quality evaluation tools (12). However, the publication of guidelines does not equate to effective clinical implementation. A survey of 116 ESGO centers in Europe showed that only 70% reported implementing ERAS protocols; even among those that did, compliance rates for operational procedures such as early urinary catheter removal (33%) and avoidance of drainage tubes (25%) remained notably low (13). The situation in China is equally concerning. Jin et al. (14) surveyed tertiary hospitals in 25 provinces and found that the implementation rates of 5 ERAS elements were below 30%, and 8 elements had rates of only 30%–70%. This gap between guidelines and practice directly impacts patients’ postoperative outcomes. Shen et al. (15) found that patients undergoing total hysterectomy with ERAS compliance ≥85% had significantly fewer postoperative complications and higher satisfaction; Jiang et al. (16) also confirmed in gynecologic oncology minimally invasive surgery that higher ERAS compliance can shorten hospital stays without increasing readmission risk.

These studies collectively suggest that improving ERAS compliance is the key pathway to better patient outcomes, and patients’ understanding of ERAS measures is an important prerequisite for compliance. Health education is the core means of establishing this cognitive foundation, and its quality directly determines whether ERAS concepts can truly be implemented. Without objective evaluation standards, improvement of health education quality has no basis. Constructing a quality evaluation scale for ERAS health education in gynecologic malignancies is precisely based on this clinical need, aiming to provide a quantifiable assessment tool for identifying weak links and driving continuous improvement in nursing quality.

### 4.2 Reliability, validity, and practicability of the scale

This study systematically examined the scale from three dimensions: content validity, construct validity, and reliability. Regarding content validity, I-CVI ranged from 0.833 to 1.000 and S-CVI/Ave was 0.946, both meeting the acceptable standards proposed by Lynn et al. (I-CVI ≥0.78, S-CVI/Ave ≥0.90) (17), indicating good content relevance between items and the measured construct. Regarding construct validity, the KMO value was 0.821 and Bartlett’s test P < 0.001; exploratory factor analysis extracted 4 common factors with a cumulative variance contribution rate of 51.60% and factor loadings of 0.569–0.796, which was highly consistent with the scale’s four-dimensional theoretical framework, indicating good construct validity. Regarding reliability, Cronbach’s α was 0.890 for the total scale and 0.805–0.902 for each dimension, all exceeding the 0.70 acceptable standard; test-retest ICC was 0.925 (95% CI: 0.788–0.964) for the total scale and 0.883–0.933 for each dimension, all >0.75, indicating good internal consistency and temporal stability.

In terms of content, this scale has three distinctive features: ① items are organized by four stages (admission, preoperative, postoperative, and discharge), highly consistent with the temporal characteristics of ERAS perioperative management, with a clear structure covering the full care continuum, facilitating stage-by-stage evaluation by clinical staff; ② ERAS-specific measures (e.g., preoperative oral carbohydrates, multimodal analgesia, early tube removal) are incorporated as independent items, distinguishing this tool from traditional health education evaluation instruments and reflecting the core connotations of ERAS health education (12); ③ items such as sexual life guidance and psychological counseling and peer support are included, addressing the psychosocial needs of gynecologic malignancy patients beyond physical recovery, reflecting the scale’s responsiveness to the specific characteristics of this population (18).

### 4.3 Clinical application value

Application of the scale to 116 perioperative patients revealed an overall scoring rate of 86.7%, with discharge education identified as the weakest dimension (84.3%) and all four items scoring below 70% concentrated in this dimension (see Results section). Beyond describing these patterns, three deeper mechanisms warrant discussion.

First, weak items cluster in domains where ERAS clinical pathways provide little behavioral specification. Items with the highest scoring rates—multimodal analgesia, early ambulation, preoperative carbohydrate loading—are precisely those embedded in standardized ERAS protocols with explicit timing and behavioral benchmarks. In contrast, low-scoring items such as post-discharge psychological support and home rehabilitation guidance lack equivalent procedural anchors, leaving nurses to rely on individual experience. This finding suggests that education quality is highly dependent on whether the underlying clinical pathway provides clear, observable behavioral standards; areas without such anchors are systematically under-addressed.

Second, the lowest-scoring items reflect culturally and emotionally sensitive topics that nurses and patients tend to avoid. Sexual recovery counseling, in particular, suffers from bidirectional avoidance—nurses fear inappropriateness, patients experience stigma—resulting in a “default referral to physicians” that rarely materializes. Similarly, post-discharge psychological needs are often acknowledged in principle but rarely operationalized through specific referral resources (counseling hotlines, peer support groups, psychiatric clinics). These findings point to the need for structured, scripted communication tools (e.g., teach-back templates, patient information leaflets) that lower the threshold for both nurses and patients to engage with sensitive topics.

Third, discharge education is consistently compressed by competing clinical priorities on discharge day. Verbal-only instructions, lack of written medication lists, and generic activity advice (“light diet,” “moderate activity”) reflect not a lack of awareness but a lack of structured delivery tools and dedicated time. International evidence (19, 20) similarly shows that unstructured discharge education contributes to medication errors, unplanned readmissions, and post-discharge anxiety.

Taken together, these findings suggest that improving discharge education requires a shift from individual nurse effort to system-level redesign: developing checklist-based discharge education packages, integrating written medication lists and family-tailored rehabilitation plans, embedding psychological referral pathways, and incorporating telephone or digital follow-up to extend education beyond hospital walls. The present scale provides a concrete tool for identifying these weak links and, more importantly, for monitoring the effectiveness of such redesign efforts through repeated quality measurement.

### 4.4 Limitations

This study has the following limitations:

1. The scale evaluates only the “health education” component of the ERAS pathway and does not encompass compliance audits of ERAS clinical measures (e.g., goal-directed fluid therapy, urinary catheter removal within 24–48 hours, intraoperative warming, multimodal analgesia execution, avoidance of routine bowel preparation). The successful implementation of ERAS relies on the synergy of clinical compliance, team adherence, patient compliance, and education quality. This scale therefore serves as a complementary evaluation tool and cannot replace comprehensive ERAS compliance audits such as the EIAS system. Future research should integrate this scale with ERAS compliance audit tools to construct a multidimensional quality framework spanning “clinical compliance—education quality—patient adherence—recovery outcomes.”
2. This study represents the scale-development and initial-application stage and did not systematically collect core ERAS outcome indicators (e.g., time to first ambulation, time to first flatus, postoperative length of stay, complication rates, 30-day readmission rates). Whether health education quality truly translates into improved patient adherence and recovery outcomes therefore could not be verified. This causal chain—”education quality → patient adherence → ERAS outcomes”—is the key evidence required to fully establish the scale’s clinical value, and warrants prospective, multicenter verification.
3. The reliability/validity testing and clinical application were conducted at a single tertiary hospital with 116 patients, limiting external generalizability.
4. Only exploratory factor analysis was performed; the stability of the factor structure should be confirmed through confirmatory factor analysis.
5. Despite the dual-perspective design combining nurse self- and patient other-evaluation, social desirability bias may persist. Future studies could incorporate independent third-party quality-control nurse ratings, video review, or in-depth patient interviews to enhance objectivity.

Future directions: The team plans to (i) integrate the scale with ERAS compliance audit tools for multidimensional evaluation; (ii) prospectively collect core ERAS outcomes and analyze associations with education quality; (iii) conduct multicenter validation; and (iv) develop standardized education intervention packages and evaluate their effects on ERAS adherence and recovery outcomes through randomized controlled trials.

## 5 Conclusion

The quality evaluation scale for ERAS health education in gynecologic malignancies developed in this study comprises 4 dimensions, 22 secondary indicators, and 36 tertiary items, demonstrating good reliability and validity. Application to 116 perioperative patients showed that the scale can effectively evaluate health education quality and identify weak links, with “psychological counseling and peer support” and “sexual life resumption guidance” emerging as the items with the lowest current scoring rates. Structured, checklist-based, and continuity-oriented discharge education represents a key direction for future improvement. Importantly, this scale serves as a dedicated evaluation tool for ERAS health education quality that complements—rather than replaces—comprehensive ERAS compliance audits. Its association with core ERAS outcomes (e.g., postoperative length of stay, complication rates, readmission rates) requires further validation through multicenter prospective studies, which represents the team’s next research priority.

## Data Availability

The datasets presented in this article are not readily available because they contain potentially sensitive patient information and are subject to institutional and ethical restrictions. Requests to access the datasets should be directed to the corresponding author, Dan Zi, at zidangy08@163.com.
