# Supplementary material for: Development and application of a quality evaluation scale for ERAS health education in gynecologic malignancies
Source: Front Oncol. 2026 Jun 30;16:1858321. doi: 10.3389/fonc.2026.1858321 (PMC13364908; doi:10.3389/fonc.2026.1858321)
Supplement: Supplementary file 1 [file Table1.docx]

Supplementary Material

# Supplementary Material S1. Quality Evaluation Scale for ERAS Health Education in Gynecologic Malignancies — Detailed Scoring Criteria for All Items

Supplementary Material S1

Supplementary Material S1 Quality Evaluation Scale for ERAS Health Education in Gynecologic Malignancies — Detailed Scoring Criteria for All Items

| First-level Dimension | Second-level Indicator | Tertiary item | Scoring Criteria | Corresponding Scores for Levels | Actual score |
| --- | --- | --- | --- | --- | --- |
| Ⅰ-1 Admission Education (30.3) |  |  |  |  |  |
|  | Ⅱ-1 Environment, Safety and Personnel Introduction (10.7) |  |  |  |  |
|  |  | Ⅲ-1 Hospital environment: ward, physician office, nursing station, hot water room (2.7) | 5 points: Provide step-by-step on-site guidance or illustrated introduction to all functional areas, enabling the patient to independently locate each area; 4 points: Introduce the main functional areas, patient has basic understanding of environment layout; 3 points: Only brief verbal explanation without on-site guidance, patient has incomplete understanding; 2 points: Only inform the location of nurse station, no other introductions; 1 point: No environmental introduction provided | □Level 5=2.7 pts □Level 4=2.16 pts □Level 3=1.62 pts □Level 2=1.08 pts □Level 1=0.54 pts |  |
|  |  | Ⅲ-2 Medical staff: attending physician, nurse, head nurse, department director (2.6) | 5 points: Fully introduce all medical staff members, provide written contact information, patient can accurately identify responsible nurse and doctor; 4 points: Introduce main responsible persons, provide basic contact information; 3 points: Only introduce some staff members without clearly providing contact information; 2 points: Only vaguely state "a nurse is responsible" without specific introduction; 1 point: No introduction of any staff members provided | □Level 5=2.6 pts □Level 4=2.08 pts □Level 3=1.56 pts □Level 2=1.04 pts □Level 1=0.52 pts |  |
|  |  | Ⅲ-3 High-risk patient identification management (wristband, bedside identification, etc.) (2.7) | 5 points: Thoroughly explain the meaning of wristbands and bedside signs, actively verify and guide patient to participate in verification; 4 points: Explain the purpose of signs, perform routine verification; 3 points: Apply wristband but do not fully explain meaning; 2 points: Only apply wristband without any explanation; 1 point: No identification management or notification provided | □Level 5=2.7 pts □Level 4=2.16 pts □Level 3=1.62 pts □Level 2=1.08 pts □Level 1=0.54 pts |  |
|  |  | Ⅲ-4 Fall/bed fall/pressure ulcer prevention (2.7) | 5 points: Complete risk assessment, provide targeted explanation of prevention measures, implement bed rails, non-slip measures and demonstrate; 4 points: Explain key prevention points, implement main protective measures; 3 points: Only verbally inform about risks, incomplete implementation of protective measures; 2 points: Only simple reminder without implementing specific measures; 1 point: No prevention education or protective measures provided | □Level 5=2.7 pts □Level 4=2.16 pts □Level 3=1.62 pts □Level 2=1.08 pts □Level 1=0.54 pts |  |
|  | Ⅱ-2 ERAS Concept Overview (2.9) |  |  |  |  |
|  |  | Ⅲ-5 ERAS concepts and benefits (2.9) | 5 points: Explain ERAS core concepts, process and benefits in simple terms with illustrated materials, patient understands and can cooperate; 4 points: Explain main content, patient has basic understanding; 3 points: Only briefly mention ERAS concept, patient has vague understanding; 2 points: Only mention the term "fast track" without explanation; 1 point: No ERAS concept education provided | □Level 5=2.9 pts □Level 4=2.32 pts □Level 3=1.74 pts □Level 2=1.16 pts □Level 1=0.58 pts |  |
|  | Ⅱ-3 VTE Prevention Management (2.8) |  |  |  |  |
|  |  | Ⅲ-6 VTE risk assessment and prevention (2.8) | 5 points: Complete Caprini or other risk assessment, provide targeted explanation and demonstration of mechanical and pharmacological prevention methods; 4 points: Complete assessment, explain main prevention measures; 3 points: Only complete assessment without adequate education; 2 points: Only briefly mention thrombosis risk; 1 point: No VTE assessment or education provided | □Level 5=2.8 pts □Level 4=2.24 pts □Level 3=1.68 pts □Level 2=1.12 pts □Level 1=0.56 pts |  |
|  | Ⅱ-4 Examination Guidance (2.7) |  |  |  |  |
|  |  | Ⅲ-7 Preoperative examination items and preparation (2.7) | 5 points: Thoroughly explain the purpose, process and key cooperation points of all examination items, patient accurately understands; 4 points: Explain main examinations and preparation key points; 3 points: Only inform about examination items without fully explaining cooperation methods; 2 points: Only verbally notify about examinations without guidance; 1 point: No examination guidance provided | □Level 5=2.7 pts □Level 4=2.16 pts □Level 3=1.62 pts □Level 2=1.08 pts □Level 1=0.54 pts |  |
|  | Ⅱ-5 Preoperative Prehabilitation (8.4) |  |  |  |  |
|  |  | Ⅲ-8 Psychological assessment and support (2.8) | 5 points: Use a scale to complete psychological assessment, provide targeted counseling and ongoing support; 4 points: Complete assessment, provide general psychological counseling; 3 points: Only briefly inquire about emotions without standardized assessment; 2 points: No assessment, only reassurance; 1 point: No attention paid to psychological status | □Level 5=2.8 pts □Level 4=2.24 pts □Level 3=1.68 pts □Level 2=1.12 pts □Level 1=0.56 pts |  |
|  |  | Ⅲ-9 Prehabilitation training (2.8) | 5 points: Develop individualized training plan, demonstrate and supervise execution, patient masters key movements; 4 points: Explain and demonstrate main training content; 3 points: Only verbally introduce training methods without demonstration; 2 points: Only mention need for training without specific guidance; 1 point: No pre-rehabilitation training guidance provided | □Level 5=2.8 pts □Level 4=2.24 pts □Level 3=1.68 pts □Level 2=1.12 pts □Level 1=0.56 pts |  |
|  |  | Ⅲ-10 Nutritional screening and intervention (2.8) | 5 points: Complete NRS2002 or other screening, provide targeted dietary guidance or nutritional support plan; 4 points: Complete screening, provide general dietary advice; 3 points: Only complete screening without intervention; 2 points: Only briefly inquire about diet without screening; 1 point: No nutritional assessment or intervention provided | □Level 5=2.8 pts □Level 4=2.24 pts □Level 3=1.68 pts □Level 2=1.12 pts □Level 1=0.56 pts |  |
|  | Ⅱ-6 Disease Knowledge (2.7) |  |  |  |  |
|  |  | Ⅲ-11 Disease knowledge, surgical plan and fertility counseling (2.7) | 5 points: Thoroughly explain disease and surgical plan, proactively provide fertility preservation consultation, patient is fully informed; 4 points: Explain disease and surgical key points, mention fertility issues; 3 points: Only explain basic disease knowledge without addressing fertility; 2 points: Only simply inform diagnosis; 1 point: No related education provided | □Level 5=2.7 pts □Level 4=2.16 pts □Level 3=1.62 pts □Level 2=1.08 pts □Level 1=0.54 pts |  |
| Ⅰ-2 Preoperative Education (25.3) |  |  |  |  |  |
|  | Ⅱ-7 Preoperative Medication and Pain Management (8.5) |  |  |  |  |
|  |  | Ⅲ-12 Purpose and method of preoperative medication (2.7) | 5 points: Thoroughly explain the name, purpose, usage and precautions of each medication, patient understands; 4 points: Explain usage and purpose of main medications; 3 points: Only inform medication name and administration time; 2 points: Only dispense medications as prescribed without explanation; 1 point: No medication guidance provided | □Level 5=2.7 pts □Level 4=2.16 pts □Level 3=1.62 pts □Level 2=1.08 pts □Level 1=0.54 pts |  |
|  |  | Ⅲ-13 Preoperative pain assessment (2.8) | 5 points: Use a standardized scale to assess pain, document and develop individualized pain management plan; 4 points: Complete assessment, provide general pain management advice; 3 points: Only inquire about pain without standardized assessment; 2 points: Only briefly mention; 1 point: No preoperative pain assessment provided | □Level 5=2.8 pts □Level 4=2.24 pts □Level 3=1.68 pts □Level 2=1.12 pts □Level 1=0.56 pts |  |
|  |  | Ⅲ-14 Multimodal analgesia regimen (3.0) | 5 points: Thoroughly explain the principles of multimodal analgesia, medications and non-pharmacological methods, patient understands and cooperates; 4 points: Explain main analgesia methods; 3 points: Only briefly introduce analgesic medications; 2 points: Only inform "painkillers will be given"; 1 point: No analgesia plan education provided | □Level 5=3.0 pts □Level 4=2.40 pts □Level 3=1.80 pts □Level 2=1.20 pts □Level 1=0.60 pts |  |
|  | Ⅱ-8 Preoperative Physical and Psychological Preparation (5.3) |  |  |  |  |
|  |  | Ⅲ-15 Personal hygiene and infection prevention (2.6) | 5 points: Provide detailed guidance on skin preparation, oral hygiene, bathing, etc., and explain key points of infection prevention; 4 points: Explain main hygiene preparation points; 3 points: Only verbally inform need to bathe, etc.; 2 points: Only remind to pay attention to hygiene; 1 point: No hygiene guidance provided | □Level 5=2.6 pts □Level 4=2.08 pts □Level 3=1.56 pts □Level 2=1.04 pts □Level 1=0.52 pts |  |
|  |  | Ⅲ-16 Emotional regulation and sleep management (2.7) | 5 points: Assess emotions and sleep, provide targeted counseling and guide relaxation and sleep aid methods; 4 points: Provide general emotional counseling and sleep advice; 3 points: Only inquire without specific guidance; 2 points: Only simple reassurance; 1 point: No attention paid to emotions and sleep | □Level 5=2.7 pts □Level 4=2.16 pts □Level 3=1.62 pts □Level 2=1.08 pts □Level 1=0.54 pts |  |
|  | Ⅱ-9 Preoperative Dietary Preparation (5.7) |  |  |  |  |
|  |  | Ⅲ-17 Purpose of preoperative oral carbohydrates (2.9) | 5 points: Thoroughly explain the ERAS rationale, dose and timing of oral carbohydrate loading, patient accurately follows; 4 points: Explain main points, patient basically follows; 3 points: Only inform administration time; 2 points: Only dispense without explanation; 1 point: No guidance provided | □Level 5=2.9 pts □Level 4=2.32 pts □Level 3=1.74 pts □Level 2=1.16 pts □Level 1=0.58 pts |  |
|  |  | Ⅲ-18 Preoperative fasting, fluid restriction and bowel preparation (2.8) | 5 points: Clearly inform fasting times according to ERAS guidelines, thoroughly explain bowel preparation methods and key cooperation points; 4 points: Inform main times and methods; 3 points: Only verbally inform fasting time; 2 points: Only simple reminder; 1 point: No guidance provided | □Level 5=2.8 pts □Level 4=2.24 pts □Level 3=1.68 pts □Level 2=1.12 pts □Level 1=0.56 pts |  |
|  | Ⅱ-10 Surgical Preparation Guidance (5.7) |  |  |  |  |
|  |  | Ⅲ-19 Surgical and anesthesia procedures (2.8) | 5 points: Use illustrations to thoroughly explain surgical and anesthesia procedures and key cooperation points, patient understands and can cooperate; 4 points: Explain main procedures; 3 points: Only brief introduction; 2 points: Only vague mention; 1 point: No procedure introduction provided | □Level 5=2.8 pts □Level 4=2.24 pts □Level 3=1.68 pts □Level 2=1.12 pts □Level 1=0.56 pts |  |
|  |  | Ⅲ-20 Postoperative tube management and early removal (2.9) | 5 points: Thoroughly explain the purpose of various tubes, care key points and significance of early removal, patient actively cooperates; 4 points: Explain main tube care key points; 3 points: Only simply inform that tubes will be placed; 2 points: Only remind not to pull; 1 point: No tube education provided | □Level 5=2.9 pts □Level 4=2.32 pts □Level 3=1.74 pts □Level 2=1.16 pts □Level 1=0.58 pts |  |
| Ⅰ-3 Postoperative Education (30.9) |  |  |  |  |  |
|  | Ⅱ-11 Positioning and Activity Guidance (5.6) |  |  |  |  |
|  |  | Ⅲ-21 Postoperative positioning and early mobilization (2.9) | 5 points: Provide time-phased guidance on position changes and early ambulation, demonstrate and supervise execution; 4 points: Explain main positioning and mobilization key points; 3 points: Only verbally inform need to mobilize; 2 points: Only simple reminder; 1 point: No positioning or mobilization guidance provided | □Level 5=2.9 pts □Level 4=2.32 pts □Level 3=1.74 pts □Level 2=1.16 pts □Level 1=0.58 pts |  |
|  |  | Ⅲ-22 Urination and pelvic floor function exercise (2.7) | 5 points: Thoroughly demonstrate pelvic floor exercise methods, guide voiding training, patient masters; 4 points: Explain main methods; 3 points: Only verbally introduce without demonstration; 2 points: Only briefly mention; 1 point: No guidance provided | □Level 5=2.7 pts □Level 4=2.16 pts □Level 3=1.62 pts □Level 2=1.08 pts □Level 1=0.54 pts |  |
|  | Ⅱ-12 Postoperative Dietary Guidance (2.9) |  |  |  |  |
|  |  | Ⅲ-23 Early feeding and dietary transition (2.9) | 5 points: Provide phased guidance on dietary transition according to ERAS guidelines, clearly specify food types and amounts; 4 points: Explain main dietary phases; 3 points: Only inform that eating is allowed; 2 points: Only simple reminder; 1 point: No dietary guidance provided | □Level 5=2.9 pts □Level 4=2.32 pts □Level 3=1.74 pts □Level 2=1.16 pts □Level 1=0.58 pts |  |
|  | Ⅱ-13 Medication and Adverse Reaction Monitoring (5.6) |  |  |  |  |
|  |  | Ⅲ-24 Postoperative medication guidance (2.8) | 5 points: Thoroughly explain the name, usage and purpose of each postoperative medication, patient understands; 4 points: Explain main medications; 3 points: Only inform administration time; 2 points: Only dispense as prescribed; 1 point: No medication guidance provided | □Level 5=2.8 pts □Level 4=2.24 pts □Level 3=1.68 pts □Level 2=1.12 pts □Level 1=0.56 pts |  |
|  |  | Ⅲ-25 Recognition and management of drug adverse reactions (2.8) | 5 points: Thoroughly explain common adverse reaction manifestations, identification and management methods; 4 points: Explain main adverse reactions; 3 points: Only briefly mention; 2 points: Only vaguely inform about side effects; 1 point: No education provided | □Level 5=2.8 pts □Level 4=2.24 pts □Level 3=1.68 pts □Level 2=1.12 pts □Level 1=0.56 pts |  |
|  | Ⅱ-14 Common Discomfort and Wound Management (5.5) |  |  |  |  |
|  |  | Ⅲ-26 Management of common postoperative discomforts (2.8) | 5 points: Thoroughly explain prevention and management methods for nausea, vomiting, bloating and other discomforts; 4 points: Explain main discomfort management; 3 points: Only simply inform; 2 points: Only reassure; 1 point: No guidance provided | □Level 5=2.8 pts □Level 4=2.24 pts □Level 3=1.68 pts □Level 2=1.12 pts □Level 1=0.56 pts |  |
|  |  | Ⅲ-27 Wound care and infection prevention (2.7) | 5 points: Thoroughly explain wound observation, dressing changes, signs of infection and prevention key points, and demonstrate; 4 points: Explain main key points; 3 points: Only verbally inform to keep clean; 2 points: Only simple reminder; 1 point: No wound education provided | □Level 5=2.7 pts □Level 4=2.16 pts □Level 3=1.62 pts □Level 2=1.08 pts □Level 1=0.54 pts |  |
|  | Ⅱ-15 Pain Management (2.9) |  |  |  |  |
|  |  | Ⅲ-28 Pain assessment and analgesia regimen (2.9) | 5 points: Use a scale to assess pain at regular intervals, implement multimodal analgesia and dynamically adjust the plan; 4 points: Complete assessment and routine analgesia; 3 points: Only inquire about pain, administer medications as needed; 2 points: Only manage when patient reports pain; 1 point: No standardized pain management provided | □Level 5=2.9 pts □Level 4=2.32 pts □Level 3=1.74 pts □Level 2=1.16 pts □Level 1=0.58 pts |  |
|  | Ⅱ-16 Complication Prevention (5.6) |  |  |  |  |
|  |  | Ⅲ-29 VTE prevention (2.9) | 5 points: Dynamically assess risk, implement mechanical and pharmacological prevention and guide early mobilization; 4 points: Implement main prevention measures; 3 points: Only verbal education; 2 points: Only simple reminder; 1 point: No VTE prevention provided | □Level 5=2.9 pts □Level 4=2.32 pts □Level 3=1.74 pts □Level 2=1.16 pts □Level 1=0.58 pts |  |
|  |  | Ⅲ-30 Lymphedema prevention and recognition (2.7) | 5 points: Thoroughly explain lymphedema manifestations, prevention methods and self-monitoring, demonstrate affected limb care; 4 points: Explain main prevention key points; 3 points: Only briefly mention; 2 points: Only vaguely inform; 1 point: No education provided | □Level 5=2.7 pts □Level 4=2.16 pts □Level 3=1.62 pts □Level 2=1.08 pts □Level 1=0.54 pts |  |
|  | Ⅱ-17 Chemotherapy Guidance (if applicable) (2.7) |  |  |  |  |
|  |  | Ⅲ-31 Chemotherapy-related symptom monitoring (2.7) | 5 points: Thoroughly explain common chemotherapy reactions, monitoring indicators and management methods, guide self-management; 4 points: Explain main reactions and management; 3 points: Only simply inform about possible reactions; 2 points: Only vaguely mention; 1 point: No chemotherapy education provided | □Level 5=2.7 pts □Level 4=2.16 pts □Level 3=1.62 pts □Level 2=1.08 pts □Level 1=0.54 pts |  |
| Ⅰ-4 Discharge Education (13.5) |  |  |  |  |  |
|  | Ⅱ-18 Home Life and Rehabilitation Guidance (2.7) |  |  |  |  |
|  |  | Ⅲ-32 Home activity, nutrition and wound care (2.7) | 5 points: Thoroughly explain home activity level, dietary nutrition and wound observation/care key points, provide written materials; 4 points: Explain main key points; 3 points: Only verbally inform precautions; 2 points: Only simple reminder; 1 point: No home guidance provided | □Level 5=2.7 pts □Level 4=2.16 pts □Level 3=1.62 pts □Level 2=1.08 pts □Level 1=0.54 pts |  |
|  | Ⅱ-19 Sexual Life Guidance (if applicable) (2.7) |  |  |  |  |
|  |  | Ⅲ-33 Sexual life resumption guidance (2.7) | 5 points: Clearly inform resumption time and precautions based on surgical situation, proactively answer questions; 4 points: Explain main key points; 3 points: Only simply inform; 2 points: Only vaguely mention; 1 point: No sexual activity guidance provided | □Level 5=2.7 pts □Level 4=2.16 pts □Level 3=1.62 pts □Level 2=1.08 pts □Level 1=0.54 pts |  |
|  | Ⅱ-20 Medication Guidance (2.7) |  |  |  |  |
|  |  | Ⅲ-34 Home medication guidance (2.7) | 5 points: Thoroughly explain the name, usage, adverse reactions and precautions of each home medication, provide written materials; 4 points: Explain main medications; 3 points: Only verbally inform administration method; 2 points: Only simply inform; 1 point: No home medication guidance provided | □Level 5=2.7 pts □Level 4=2.16 pts □Level 3=1.62 pts □Level 2=1.08 pts □Level 1=0.54 pts |  |
|  | Ⅱ-21 Follow-up Arrangements (2.8) |  |  |  |  |
|  |  | Ⅲ-35 Follow-up plan and contact information (2.8) | 5 points: Clearly inform follow-up time, items, location and contact information, provide written appointment information; 4 points: Inform main follow-up arrangements; 3 points: Only verbally inform follow-up time; 2 points: Only vaguely mention; 1 point: No follow-up plan provided | □Level 5=2.8 pts □Level 4=2.24 pts □Level 3=1.68 pts □Level 2=1.12 pts □Level 1=0.56 pts |  |
|  | Ⅱ-22 Psychological Support (2.7) |  |  |  |  |
|  |  | Ⅲ-36 Psychological counseling and peer support (2.7) | 5 points: Assess post-discharge psychological status, provide access to psychological counseling and patient peer support resources; 4 points: Provide main psychological support information; 3 points: Only verbal encouragement; 2 points: Only simple reassurance; 1 point: No psychological support provided | □Level 5=2.7 pts □Level 4=2.16 pts □Level 3=1.62 pts □Level 2=1.08 pts □Level 1=0.54 pts |  |
